# Lack of Ser267Phe variant of sodium taurocholate cotransporting polypeptide among Moroccans regardless of hepatitis B virus infection status

**DOI:** 10.1186/s12879-017-2214-2

**Published:** 2017-01-26

**Authors:** Sayeh Ezzikouri, Hajar Chihab, Abdellah Elhabazi, Lahcen Wakrim, Soumaya Benjelloun

**Affiliations:** 1 Virology Unit, Viral Hepatitis Laboratory, Institut Pasteur du Maroc, 1, Place Louis Pasteur, 20360 Casablanca, Morocco; 2 Cell Biology Department, Faculty of Sciences, Chouaib Doukkali University, El-Jadida, Morocco; 3 Virology Unit, Immunovirology Laboratory, Institut Pasteur du Maroc, Casablanca, Morocco

**Keywords:** Hepatitis B virus, Functional receptor, NTCP, Polymorphism

## Abstract

**Background:**

The sodium taurocholate co-transporting polypeptide, encoded by SLC10A1, was identified as a functional receptor for hepatitis B virus (HBV). The objective of this study was to determine if there was an association of the Ser267Phe variant (rs2296651) with HBV infection status in Moroccan patients.

**Methods:**

Using a TaqMan 5’ allelic discrimination assay, the Ser267Phe variant was genotyped in 286 chronic hepatitis B patients, 135 individuals with spontaneous clearance from HBV infection and 109 healthy controls negative for hepatitis B serological markers.

**Results:**

In this cohort, we detected only wild-type genotype (S267S) in all groups. This polymorphism was not associated with the HBV infection status in Moroccan patients.

**Conclusions:**

The S267F variant is absent among Moroccans regardless of chronic HBV infection status.

## Background

Chronic hepatitis B (CHB) affects about 248 million people worldwide and can lead to liver cirrhosis, liver failure and hepatocellular carcinoma, especially in many Asian and African countries [1, 2]. Whereas around 90% of the HBV-infected adults develop acute infection followed by spontaneous viral clearance, 5–10% of infected individuals are not able to clear the virus and develop chronic infection [3, 4]. Morocco is a country of intermediate endemicity of hepatitis B virus (HBV) and diverse modes of transmission including vertical, intra-familial, sexual or parenteral have been reported [5]. Several studies have highlighted that host genetic factors could influence the course of the disease [6, 7]. Recently, Yan and colleagues identified sodium taurocholate cotransporting polypeptide (NTCP) as a functional receptor of HBV [8]. NTCP is a member of the solute carrier family 10 (SLC10), the major bile acid uptake system in human hepatocytes, and localized to the basolateral hepatocyte membrane [9]. Silencing the NTCP expression in primary *Tupaia* hepatocytes, HepaRG or primary human hepatocytes diminished HBV infectivity [8]. A genetic polymorphism (S267F, c.800G > A, rs2296651) in the solute carrier family 10 member 1 gene (*SLC10A1*) completely abolished the HBV receptor function of human NTCP [10]. In addition, a previous study showed that the S267F mutant of NTCP had normal cell surface expression, but resulted in almost complete loss of bile salt uptake [11]. Starting from this known functional polymorphism, some case control association studies on HBV infection were conducted among Asian patients. However, the results were not consistent [12–15].

The aim of this study was to determine whether the *SLC10A1* rs2296651 (S267F) polymorphism was associated with resolution or the progression of hepatitis B in patients from Morocco.

## Methods

To address this issue, a total of 530 consented Moroccan participants were independently recruited at Institut Pasteur du Maroc. From May 2008 through January 2015, 286 CHB patients (172 males) with a median age of 42 years (range 19–78), 135 (52 males) individuals with spontaneous clearance from HBV infection, who were positive for antibody against hepatitis B core antigen and negative for hepatitis B surface antigen (HBsAg), with a median age of 52 years (range 19–85) and 109 healthy controls (55 males) negative for hepatitis serological markers with a median age of 53 years (range 25–76) were enrolled in the present study. The study was approved by the Ethics Committee of the Faculté de Medicine of Casablanca. The rs2296651 polymorphism was genotyped using a TaqMan SNP Genotyping Assay (Applied Biosystems; assay ID C_16184554_10). Approximately 15% of total DNA samples were repeated for quality control, and no discrepancy was observed.

## Results & discussion

We investigated the association between the serine/phenylalanine polymorphism located at codon 267 of *SLC10A1* and HBV infection status in a case-control study of 286 CHB and 135 subjects with spontaneous clearance from HBV infection. Moreover, to estimate the frequencies of the *SLC10A1* genotypes in the general Moroccan population, the rs2296651 was genotyped in 109 healthy controls. The demographic, biochemical and virological characteristics of the population studied are shown in Table 1. In this cohort, we detected only wild-type genotype (S267S) in all groups and there were no differences between groups. Our finding is in contrast with previous asian studies, and suggest that this polymorphism may be specific to Asian populations. A recent large cohort study showed that heterozygous and homozygous subjects for S267F polymorphism were resistant to CHB [12]. Moreover, other findings support this association and showed that S267F homozygotes were strongly associated with HBsAg-seronegativity [13]. However, other previous studies showed that the S267F variant was associated with susceptibilty to HBV infection [14, 16]. Furthermore, a recent report found that the frequency of the mutant allele was decreased in the positive HBsAg group and increased in HBsAg and HBeAg positive groups relative to controls [15].

**Table 1:** Baseline characteristics of patients

	Chronic HBV infection (*N* = 286)	HBV-Spontaneous clearance (*N* = 135)	Healthy controls (*N* = 109)
Median age (range), y	42 (19–78)	52 (19–85)	53 (25–76)
Gender (%)			
Male	60.14	38.51	50.46
Female	39.86	61.49	49.54
Mean Alanine aminotransferase ± SD (IU/L)	55.66 ± 25.63	28.36 ± 6.32	26.80 ± 5.72
Mean Aspartate aminotransferase ± SD (IU/L)	48.09 ± 24.87	25.93 ± 5.45	21.70 ± 2.64
HBeAg positive (%)	5.5	–	–
Anti-HBe positive (%)	94.5	–	–
Median log10 HBV-DNA (IU/ml)	4.4 (3.8-6.5)	–	–
Genotypes (%)			
D	89	–	–
A	11	–	–
*SLC10A1* (rs2296651, G > A)			
GG	286 (100%)	135 (100%)	109 (100%)
AG	0	0	0
AA	0	0	0

## Conclusions

Our data showed that S267F is not associated with HBV infection, and is not prevalent in the general Moroccan population.

## Abbreviations

CHB: Chronic hepatitis B
HBV: Hepatitis B virus
NTCP: Sodium taurocholate cotransporting polypeptide
SLC10A1: Solute carrier family 10 member 1
